# Exposing to Cadmium Stress Cause Profound Toxic Effect on Microbiota of the Mice Intestinal Tract

**DOI:** 10.1371/journal.pone.0085323

**Published:** 2014-02-03

**Authors:** Yehao Liu, Yuhui Li, Kaiyong Liu, Jie Shen

**Affiliations:** 1 School of Public Health, Anhui Medical University, Hefei, Anhui Province, People′s Republic of China; 2 Department of Biological and Environmental Engineering, Hefei Univeristy, Hefei, Anhui Province, People′s Republic of China; University of Chicago, United States of America

## Abstract

Cadmium (Cd), one of the heavy metals, is an important environmental pollutant and a potent toxicant to organism. It poses a severe threat to the growth of the organism, and also has been recognized as a human carcinogen. However, the toxicity of cadmium and its influences on microbiota in mammal's intestine are still unclear. In our experiment, the changes of intestinal microbiota in two groups of mice were investigated, which were supplied with 20 and 100 mg kg^−1^ cadmium chloride respectively for 3 weeks. The control group was treated with water free from cadmium chloride only. This study demonstrated that Cd accumulated in some tissues of mice after Cd administration and the gut barrier was impaired. Cd exposure also significantly elevated the colonic level of TNF-α. On the other hand, Cd-treatment could slow down the growth of gut microbiota and reduced the abundance of total intestinal bacteria of the mice. Among them, the growth of Bacteroidetes was significantly suppressed while Firmicutes growth was not. The probiotics including Lactobacillus and Bifidobacterium were notably inhibited. We also observed that the copies of key genes involved in the metabolism of carbohydrates to short-chain fatty acids (SCFAs) were lower in Cd-treated groups than control. As a result, the levels of short-chain fatty acids in colonic decreased significantly. In summary, this study provides valuable insight into the effects of Cd intake on mice gut microbiota.

## Introduction

Cadmium is a toxic heavy metal with a variety of sources in the environment and from industry including use in electroplating, paint and mining [1]. It is also associated with waste water pollution, and its discharge into water and food resulting in adverse effects on living organisms and the environment [2]. Cadmium has a long biological half-time of 10–30 years in human kidney. Even chronic low levels of Cd can lead to renal failure, deregulated blood pressure, diabetic complications and it also affects bone structure thereby leading to osteoporosis [3]. Moreover, Cd is also associated with airway inflammation, cardiovascular diseases, diabetes, neurological diseases and several cancers [3], [4], [5].

The gastrointestinal (GI) tract acts as the first organ susceptible to the xenobiotics. The normal microflora comprises diverse populations of bacteria and has mutual relationship with intestinal epithelial cells [6]. They are known to live and in symbiosis and play an essential role in the development and health of the host by improving the intestinal tract microbial balance as well as detoxification and elimination of poisonous compounds from the body by removing metals through precipitation and other ways [7]. The indigenous GI tract microflora has profound effects on the physiological and immunological development of the host. The intestinal microflora of mammals is involved in host nutrition. The presence of commensal bacteria in the intestinal tract also provides the first barrier of defense against pathogenic bacteria [8]. The indigenous microflora stimulates the host immune system to respond more quickly to outer challenges. It is well known that the imbalance in the relationship between intestinal epithelial cells and bacteria results in GI disorders [9]. However, early studies of the effects of xenobiotics on gut microbiota were limited by the use of culture-based technologies that identified <5% of the extant GI tract microbes. Culture-independent investigation of ribosomal RNA sequences allows the microbial population and structure of the gut microbiota to be profiles with greater resolution [10].

Toxicants, including heavy metals and pathogens reach intestine following ingestion of contaminated food and water, and interact with an ecosystem of eukaryotic and prokaryotic cells [11]. Since microorganisms play a major role in the host homeostasis, the effect of heavy metal toxicity on gut microflora has received attentions in recent years [12], [13]. However, the toxicological effect of heavy metals, especially Cd on GI microflora, is still remains unclear. The present study explores the toxic effects of Cd on the changes of intestinal bacteria quantity and SCFAs metabolism.

## Materials and Methods

### Chemicals

Cadmium chloride (CdCl_2_, MW = 183.03) analytical grade was purchased from Merck, Germany. Cadmium solutions with varying concentrations of CdCl_2_ were prepared in distilled water.

### Animals

Animal studies and experiments were approved and carried out according to Anhui Medical University's Standing Committee on Animals. Thirty healthy female Balb/c mice aged 55 to 60 days were used. Mice were kept on 12-hour reversed light/dark cycle and the temperature was maintained at 22°C in the animal house facility and pellet diet and water were supplied.

### Experimental design

The study was carried out using a randomized design involving 30 mice. The duration of the study was consisting of a 21-day treatment followed by a 7-day interval of sample collection.

### Administration of cadmium chloride

Mice were randomly divided into three groups consisting 10 mice each. Group 1 was used as control which was given cadmium-free distilled water. Group 2 was fed with the cadmium chloride with the final concentration of 20 mg kg^−1^ (low concentration Cd); Group 3 was supplied cadmium chloride with the final concentration of 100 mg kg^−1^ (high concentration Cd).

### Culturing of fecal microbiota

Fresh fecal material was placed in PBS and suspended by overtaxing for 5 min, and the suspension was allowed to stand at room temperature for 5 min to permit large insoluble particles to settle to the bottom of the tube. The supernatant was inoculated with needle to the plates 150 mm in diameter containing nonselective Gut Microbiota Medium so that the diameter of colonies was measured. The plates were incubated at 37°C under an atmosphere of 75% N_2_, 20% CO_2_, and 5% H_2_ [14].

### Tissue collection, preparation and cadmium measurements

Body weight of the mice was determined prior to sacrifice by decapitation. Two different digestion procedures were carried out: one for whole blood samples and another for mice tissue samples. Fifty microliters of whole blood were wet digested with 500 µL of 3% nitric acid at 65°C during 1 h in a plastic digestion vessel on a block heater. Freshly excised pieces of tissue samples (liver, kidney and small intestine) were collected and snap-frozen in liquid nitrogen for cadmium analysis.

The Cd concentration was measured by atomic absorption spectrometry after tissue sample preparation as described by Hofer et al. [15]. In brief, pieces of tissue were dried for 4 h at 60°C. 65% HNO_3_ was added to lyophilized tissue samples, digested with a high performance microwave system. A solution prepared from digested sample and bi-distilled water was used for determination of cadmium by graphite furnace atomic absorption spectrometry.

### Measurement of mucus layer thickness

Proximal colon segments were immediately removed and fixed in Carnoy's solution (ethanol 6: acid acetic 3: chloroform 1, vol/vol) for 2 h at 4°C. They were then immersed in ethanol 100% for 24 h. Paraffin sections of 5 µm were stained with hematoxylin-eosin. A minimum of 20 different measurements were made perpendicular to the inner mucus layer per field. Five randomly selected fields were analyzed for each colon by using an image analyzer [16].

### Measurement of colonic cytokine TNF-α

The level of TNF-α in colon tissue was measured by enzyme-linked-immunosorbent assay using commercial CoWin TNF-α ELISA kit (CoWin Biosciences, China). Briefly, the colons were collected after washing in cold phosphate-buffered saline, and then homogenized in extraction buffer (EB) containing protease inhibitor in 50 mL, 100 mM phosphate buffer (100 mg tissue per mL EB). The homogenized colon tissue was centrifuged on 2000 rpm at 4°C for 15 min. Cytokine concentration was determined in the supernate according to the manufacturer's instruction.

### Gas chromatographic analysis of SCFAs

Mouse fecal pellets were collected at week 1, 2 and 3 and frozen until analyzed. Single pellets were weighed and homogenized in 100 µL of deionized water for 3 min. The pH of the suspension was adjusted to 2–3 by adding 5 M HCl at room temperature for 10 min with intermittent shaking. The suspension was transferred into a polypropylene tube and centrifuged for 20 min at 3,000 g, yielding a clear supernatant. The internal standard, 2-ethylbutyric acid (TEBA), was added into the supernatant at a final concentration of 1 mM. Chromatographic analysis used the Agilent 7890 (Agilent). A fused-silica capillary column (30 m, 0.52 mm, 0.50 mm) with a free fatty acid phase (DB-FFAP 125-3237, J&W Scientific, Agilent Technologies Inc.) was used for analysis. Helium was the carrier at a flow rate of 14.4 mL min^−1^. The initial oven temperature (100°C) was maintained for 30 s, raised to 180°C at 8°C min^−1^ and held for 60 s, then increased to 200°C at 20°C min^−1^ and held for 5 min. The flame ionization detector and injection port were kept at 240 and 200°C, respectively. The flow rates of hydrogen, air, and nitrogen were 30, 300 and 20 mL min^−1^, respectively. The injected sample volume for GC analysis was 1 µL, and each analysis had a run time of 32 min [17].

### DNA extraction and quantitative PCR amplification

DNA extractions from fecal pellets were performed using the Sangon DNA stool extraction kit (Sangon, China) according to the manufacturer's protocol. Total extracted DNA was quantified using Nanodrop 1000 (Thermo Scientific). PCR to confirm bacterial DNA extractions was performed using the 27F/1492R bacterial primers for 16S rRNA.

After genomic DNA extraction and quantification, samples were prepared for amplification. Quantitative PCR assays were applied to assess for taxa of interest were performed on a Roche 480 quantitative PCR cycler using the UltraSYBR Mixture kit (Cowin, China) according the manufacture's instructions. All primer sequences are provided in Table 1.

**Table 1:** Primers used for real-time quantitative PCR for taxonomic and functional analyses.

Primer	Forward sequence	Reverse sequence
16S rDNA	AGAGTTTGATCCTGGCTCAG	TACCTTGTTACGACTT
*Bacteroidetes*	GGARCATGTGGTTTAATTCGATGAT	AGCTGACGACAACCATGCAG
*Firmicutes*	GGAGYATGTGGTTTAATTCGAAGCA	AGCTGACGACAACCATGCAC
BCoAT	GCIGAICATTTCACITGGAAYWSITGGCAYATG	CCTGCCTTTGCAATRTCIACRAANGC
FTHFS	GTWTGGGCWAARGGYGGMGAAGG	GTATTGDGTYTTRGCCATACA
*Bifidobacterium*	TCGCGTC(C/T)GGTGTGAAAG	CCACATCCAGCRTCCAC
*Lactobacillus*	AGCAGTAGGGAATCTTCCA	CACCGCTACACATGGAG

Specific degenerate (R = A or G; W = A or T; M = A or C; Y = C or T; D = A, G, or T; I = inosine; N = A, C, G, or T) primers were used to assess for taxa of interest, including Bacteria, Bacteroidetes and Firmicutes. Degenerate primers were used to assess for two genes involved in short-chain fatty acid synthesis, butyryl CoA transferase (BCoAT) and formyltetrahydrofolate synthetase (FTHFS).

### Statistical analysis

Values are expressed as the mean ± SD of triplicates. Statistical analysis was analyzed using one-way ANOVA. Statistical significance was considered as *p*<0.05.

## Results

### Cd exposure inhibited the growth rate of bacteria populations

**Figure 1:**
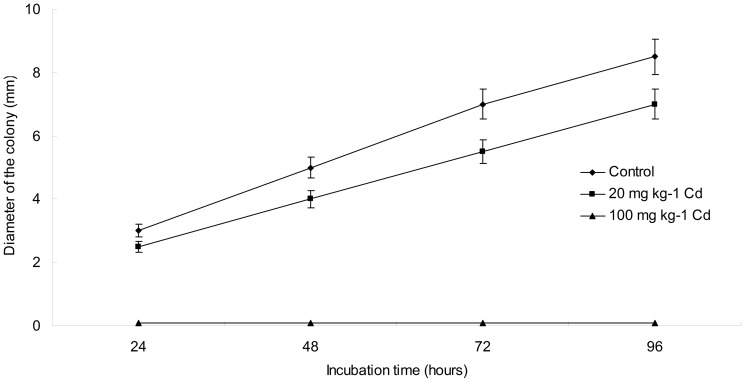
Comparison of the diameter of the colony between control and Cd treatments during the period of incubation.

The growth rate of bacterial populations was evaluated using intestinal secretion and biopsy cultures for total bacteria (Fig. 1). After been incubated at 37°C for 24 hours, the growth rate was inhibited significantly by Cd treatments, which was reflected by diameter of bacterial colony. The increase of bacterial colony diameter was shown in figure 1 during the period of 96 hours incubation. There was no growth for bacteria under the treatment of high concentration of Cd. The diameter of bacterial colony was reduced significantly 16.7% under the treatment of low concentration of Cd when compared to the control.

### Cd concentration increased in the tissue samples of mice

The analysis of Cd concentrations in the tissue samples revealed dose-related increase in Cd levels. The concentration of Cd increased significantly in all samples during the period of experiment (Table 2). Two daily doses of Cd by drinking water resulted in the highest Cd level in kidney sample, the lowest Cd level in blood sample.

**Table 2:** Cd content in blood, liver, kidney and colon of mice during the period of experiment.

		Control	20 mg kg^−1^	100 mg kg^−1^
Blood (µg.L−1)	Week 1	0	6.10±0.47	25.60±1.97
	Week 2	0	14.73±1.11	49.21±4.32
	Week 3	0	23.12±1.65	75.35±5.79
Liver (mg.kg−1)	Week 1	0	0.64±0.06	2.62±0.23
	Week 2	0	1.92±0.15	8.64±0.71
	Week 3	0	3.50±0.27	14.34±1.09
Kidney(mg.kg−1)	Week 1	0	1.12±0.09	4.69±0.37
	Week 2	0	4.58±0.33	20.10±1.56
	Week 3	0	7.40±0.57	31.52±2.47
Colon(mg.kg−1)	Week 1	0	0.54±0.04	2.51±0.22
	Week 2	0	2.13±0.15	5.73±0.48
	Week 3	0	4.81±0.39	12.14±0.97

Data were mean±SD.

### Cd treatment decreased the thickness of inner mucus layer

**Figure 2:**
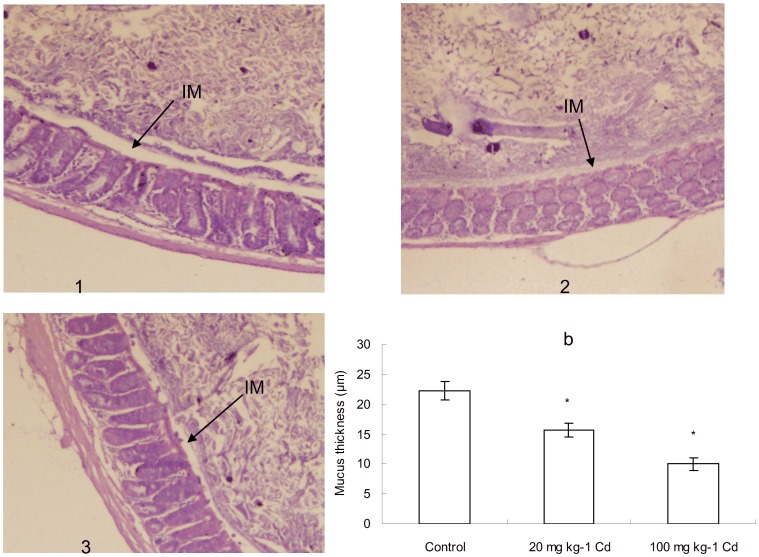
Representative HE images that were used for mucus layer thickness measurements (n = 4). 1 from the control, 2 from 20^−1^ Cd, 3 from 100 mg kg^−1^ Cd. IM, inner mucus layer, (A). Thickness of the mucus layer measured by histological analyses after HE staining. Data with asterisk were significantly different (*p*<0.05), (B).

Recent researches indicate that the interactions between the gut microbiota and mucus layer are dynamic systems which could affect mucus biology. Therefore, we investigated the impact of Cd treatment on the thickness of the inner mucus layer (Fig. 2a, 2b). We demonstrated a decrease in mucus layer in Cd-treated mice.

### The level of TNF-α in colon increased after intake of cadmium

**Figure 3:**
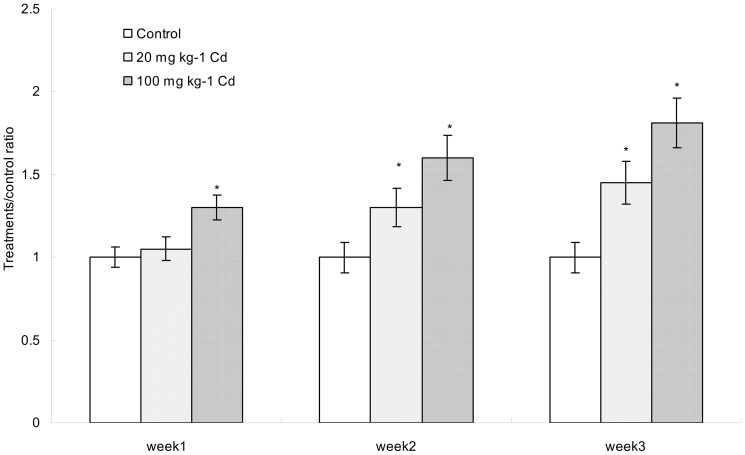
The comparison of TNF-α during the period of experiment. Data with asterisk were significantly different (p<0.05).

As shown in Figure 3, oral administration of mice with cadmium-polluted water caused a significant increase in colon level of TNF-α as compared to the control group. The level of TNF-α increased significantly at week 1 in the high Cd-treated group. Moreover, oral administration of low and high concentrations of Cd for 2 and 3 weeks led to a remarkable elevation in the level of TNF-α as compared to that of the control group.

### Cd exposure altered overall gut microbial census and the composition of intestinal microbiota

**Figure 4:**
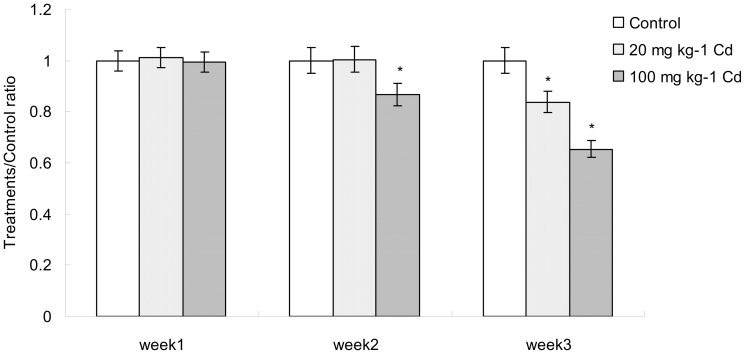
The comparison of total bacterial census during the period of experiment. Data with asterisk were significantly different (p<0.05).

To determine whether Cd exposure affected the intestinal microbiome, microbial DNA extracted from fecal samples of mice were studied (Fig. 4). Determined through quantitative PCR using 341F/518R universal primers, the census in the Cd-treated and control mice showed no significant difference at week 1. However, the amount of total bacteria decreased significantly under the treatment of high concentration of Cd at week 2. Moreover, quantity of total bacteria in low and high concentrations of Cd-treated samples decreased significantly at week 3 when compared to control. These data indicated that Cd exposure could cause substantial changes in the overall microbial census.

**Figure 5:**
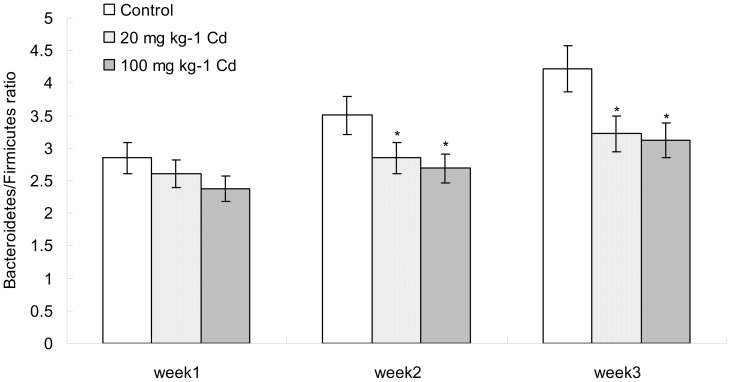
The comparison of Firmicutes/Bacteroidetes ratio during the period of experiment. Data with asterisk were significantly different (*p*<0.05).

We also assessed the composition of the microbial populations in fecal samples, (Fig. 5). The ratio of Firmicutes to Bacteroidetes showed no difference between treatments and control at week 1. But at weeks 2 and 3, the ratio of Firmicutes to Bacteroidetes decreased significantly both in low and high Cd treatments when compared with control.

**Figure 6:**
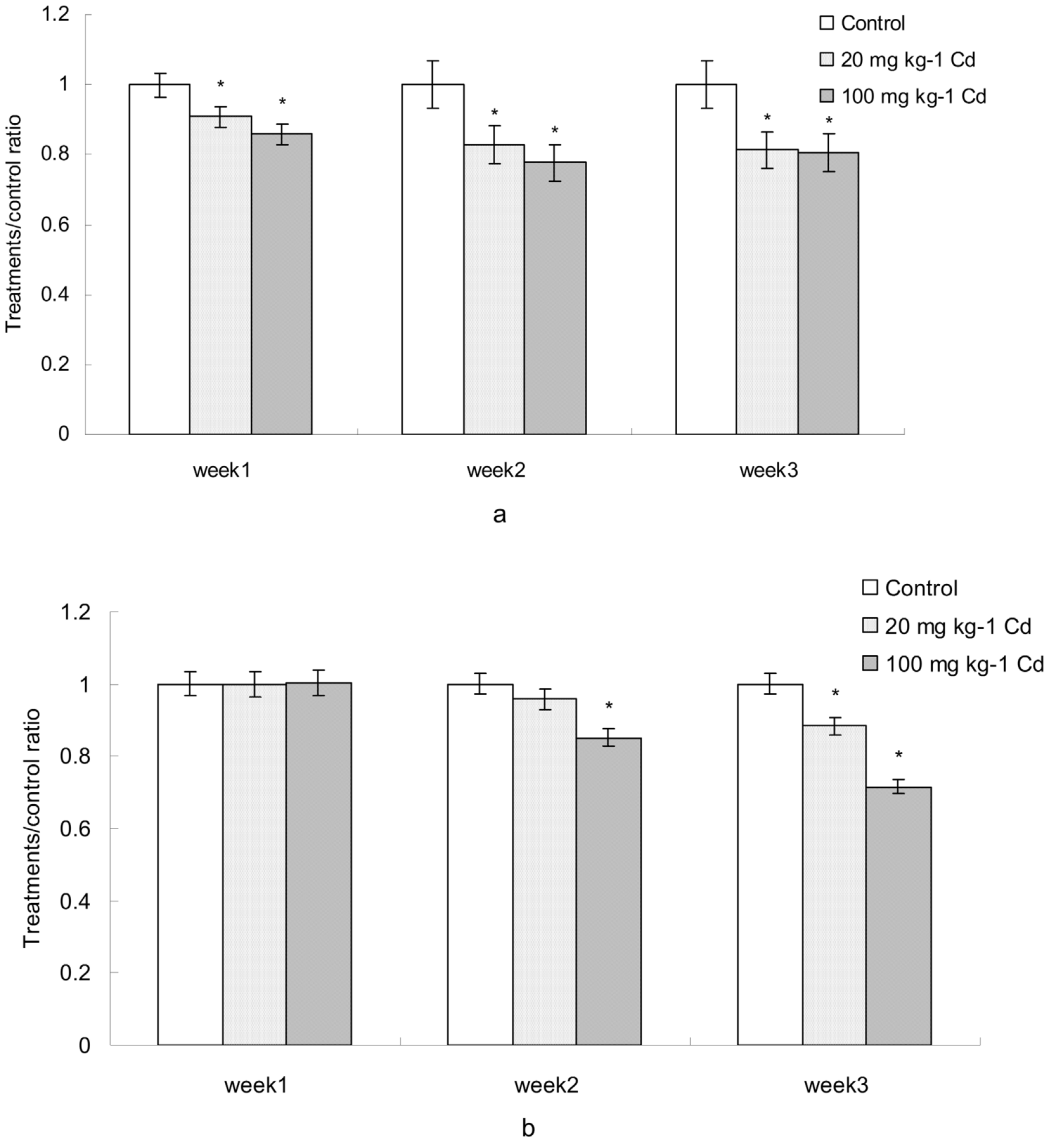
The comparison of probiotics during the period of experiment. a, *Bifidobacteria*; b, *Lactobacilli*. Data with asterisk were significantly different (*p*<0.05).

Probiotics such as Lactobacilli and Bifidobacteria can provide specific health benefit for their host. It is necessary to evaluate whether they were harmed by Cd exposure. During the period of experiment, the population of Bifidobacteria was decreased significantly by Cd treatment compared to control (Fig. 6a). In contrast, the population of Lactobacilli was harmed by high concentration of Cd at week 2 and 3. Meanwhile, population of Lactobacilli decreased significantly under the stress of low concentration of Cd at week 3 (Fig. 6b).

### Exposure to Cd altered gut microbiome SCFAs metabolism

Because of the central role of SCFAs synthesis in colonic metabolism, we examined the effect of Cd exposure on gene counts of prokaryotic genes butyryl coA transferase (BCoAT) and formyltetrahydrofolate synthetase (FTHFS) that are involved in butyrate and acetate synthesis respectively. Degenerate qPCRs for BCoAT and FTHFS were performed on fecal specimens from control and Cd-treated mice (Table 3). At week 1, there were no significant changes in BCoAT gene copy number. But at week 2 and 3, BCoAT copy numbers had decreased significantly in all treatments when compared to control. For FTHFS, there were no significant differences between control and treatments at week 1 and 2. However, significant decrease appeared in treatments at week 3 when compared to control.

**Table 3:** The result of qPCR for butyryl CoA transferase (BCoAT) and formyltetrahydrofolate synthetase (FTHFS) genes.

	BCoAT (For butyrate)			FTHFS (For acetate)		
	week 1	week 2	week 3	week 1	week 2	week 3
Control	0.046±0.007	0.056±0.006	0.081±0.006	0.35±0.02	0.42±0.02	0.56±0.02
20 mg kg^−1^ Cd	0.036±0.004	0.041±0.005	0.067±0.004	0.34±0.03	0.41±0.03	0.46±0.02
100 mg kg^−1^ Cd	0.034±0.004	0.039±0.003	0.065±0.002	0.35±0.04	0.38±0.01	0.39±0.02
*p* value	0.06	0.006	0.008	0.96	0.08	0.0002

Values were the percentage of BCoAT copies/Bacteria copies and FTHFS copies/Bacteria copies.

**Figure 7:**
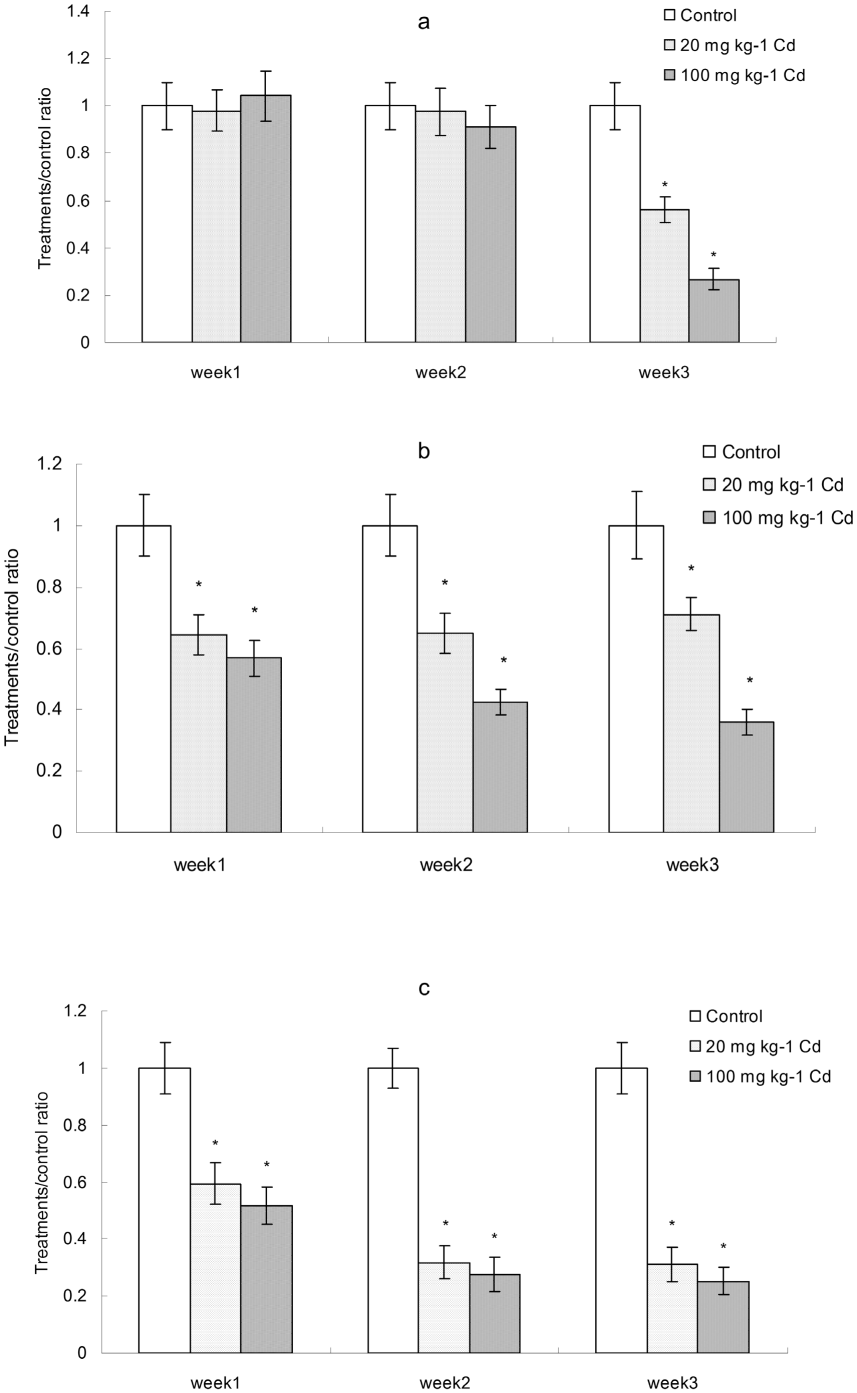
SCFAs concentration analyzed by gas chromatography (GC). a, acetate; b, butyrate; c, propionate. Data with asterisk were significantly different (*p*<0.05).

Direct measurements of SCFAs in the fecal contents of control and Cd-treated mice demonstrated substantial decreases in acetate, propionate and butyrate. Specifically, the amount of acetate was highest, while that of butyrate was lowest showed by GC (data not shown). There was no significant decrease in acetate concentration at weeks 1 and 2 between Cd treatments and control. However, the concentration of acetate decreased significantly in treatments at week 3 (Fig. 7a). Compared to acetate, the concentrations of propionate and butyrate were significantly lower than control during the period of experiment (Fig. 7b, 7c). These findings provided the evidence that Cd exposure disturbed the metabolic capabilities of the microbiome, specifically with respect to SCFAs.

## Discussion

Here, we used a murine model to gain insight into the toxicity of Cd to intestinal microbiota. In this study, we focused on the microbiota and its response to host consumption of water containing Cd. We found that the growth rate of intestinal microbiota was inhibited significantly in vitro under Cd stress. Gut barrier was impaired as a result of Cd accumulation in intestine. Probiotic Bifidobacteria and Lactobacilli received more stress from Cd than other components of gut bacteria. Cd intake resulted in a decrease of butyrate-producing bacteria, which leads to the increase in cecal pH, and decrease in fecal SCFAs. This study provides a gut microbe-based framework for evaluating responses to Cd intake.

The existence of heavy metals in living organisms can generate different degrees of adverse effects on liver, brain, intestinal systems and *et.al.* [1], [18]. For example, previous data suggest that heavy metals contribute to gut barrier alterations because intestine is the main absorbing section [19]. However, the different mechanisms of the interaction between heavy metals and the host that affect gut barrier function have not been fully elucidated. We determined Cd levels for different duration of exposure. As shown in this study, administration of CdCl_2_ resulted in clearly elevated Cd level in some tissue samples. Since the accumulation of Cd, the histological structure and function of intestine could be harmed, definitely including the intestinal microbiota. However, different effects of Cd toxicity would be exhibited according to the exposure duration and dosage to Cd. This study identified an association of Cd treatment with a decrease in mucus thickness, which supports a mechanism of increased gut permeability. Furthermore, various concentrations of heavy metals have demonstrated to be growth limiting or retarding against diverse microorganisms [20], [21]. The toxic effects of heavy metals on gut microbiota impose great impact on food digestion as well as host health [12], [13]. This study has examined the effect of cadmium chloride on the growth rate of mice intestinal microbiota in vitro. The result demonstrated that the growth rate of intestinal microbiota was retarded noticeably even under the low concentration of Cd. This may result from its high ability in inducing oxidative stress via indirect mechanisms.

SCFAs, in particular butyric acid, is a molecule of interest as dietary fiber degraded by microbes led to increased levels of butyrate and butyrate-producing commensal anaerobes. This may result from metabolic from resident butytate-producing Firmicutes [22]. Since the toxic effect of cadmium on the intestinal microbiota, their population was severely reduced. This might lead to the low butyrate-producing commensal anaerobes and the expression of BCoAT gene. In addition to butyric acid, propionic and acetic acid were also decreased in the fecal contents of Cd-treated mice. This might be similar to the pattern of butyrate acid. One of the key functions of SCFAs is maintaining acidic conditions in intestine [23]. The cecal pH may increase as a result of SCFAs decrease. It may create favorable conditions for opportunistic pathogen and pathogens. On the other hand, the SCFAs in the colon are important nutrients for the mucosal cells and may stimulate the proliferation of the coloncyctes and increase the blood flow [24], [25]. The decrease of SCFAs concentrations may deteriorate intestinal physiological function.

Probiotic Bifidobacteria and Lactobacilli can benefit both microbial and host physiology [7]. Certain Bifidobacteria may influence Enterobacteriaceae by decreasing their virulence gene expression, and the expression patterns of the Salmonella pathogenicity islands SPI1 and SPI2 [26], [27]. Probiotic strains may also exert direct effects on the host mucosa [16]. Oxidative stress is a key feature of Cd toxicity to organism, and the decrease of probiotic measured by qPCR may deteriorate this oxidative stress as indicated in this study. However, our result showed that Bifidobacteria was more sensitive to the toxicity of Cd than Lactobacilli. Two factors may contribute to this phenomenon. First, our qPCR result and other study showed that the population of Lactobacilli was about 100 folds higher than that of Bifidobacteria. A large number of Lactobacilli might have more resistance to the toxicity of Cd than *Bifidobacteria* [28]. Second, Bhakta's research showed that Lactobacilli have a excellent capability in heavy metal removal [29].

In summary, this study provided several substantial insights to illustrate the toxicity of cadmium to mice gut microbiota. Cd treatment could decrease the population of gut bacteria remarkably especially the probiotics in a short period of time. The thickness of mice inner mucus layer was also attenuated by Cd treatment. The concentrations of SCFAs from gut friendly bacteria dropped as a result of Cd toxicity. These results widen our knowledge about the toxicity of Cd.
